# Editorial: Modulating Vascular Lymphatic Growth in Disease: Current and Potential Pharmacological Approaches for Prevention and Treatment

**DOI:** 10.3389/fphar.2022.910142

**Published:** 2022-04-25

**Authors:** Ines Martinez-Corral, Natalie L. Trevaskis, Melissa García-Caballero

**Affiliations:** ^1^ Laboratory of Development and Plasticity of the Neuroendocrine Brain, Lille Neuroscience & Cognition, Inserm UMR-S1172, University of Lille, Inserm, CHU Lille, Lille, France; ^2^ Department of Drug Delivery, Disposition and Dynamics, Monash Institute of Pharmaceutical Sciences, Monash University, Melbourne, VIC, Australia; ^3^ Department of Molecular Biology and Biochemistry, Faculty of Sciences, University of Málaga, Andalucía Tech, Málaga, Spain; ^4^ IBIMA (Biomedical Research Institute of Málaga)-Plataforma BIONAND, Málaga, Spain

**Keywords:** lymphatic vessel, lymphedema, cancer, obesity, metabolic disorders, treatment

The lymphatic system is part of the circulatory system and it is indispensable for life. In physiological conditions, the main functions assigned to the lymphatic system are the maintenance of the interstitial fluid homeostasis, immune surveillance and the absorption of dietary fat in the intestine (Alitalo, 2011). However, morphological or functional changes in lymphatic vessels can contribute to disorders such as lymphedema, tumor metastasis, inflammation and other pathological conditions (Oliver et al., 2020). Therefore, there is an urgent need to further understand the mechanisms driving lymphatic dysfunction in these conditions and to develop novel lymph-targeted therapies. In this Research Topic, we present a collection of research articles that provide new insights into obesity-induced lymphatic dysfunction and tumor-draining lymph node reconstruction; and review articles that highlight and discuss the latest pharmacological approaches to treat lymphedema and mechanisms to target obesity and metabolic diseases through modulating lymphatic contraction.

Primary (or genetic) and secondary (or acquired) lymphedema are characterized by skin and tissue edema, chronic swelling, inflammation and fibro-adipose tissue accumulation in the extremities due to abnormal lymphatic fluid clearance (Rockson, 2021). Approximately 10 million people in the United States and hundreds of millions of individuals worldwide suffer from lymphedema. A comprehensive review by Brown et al. summarizes potential pharmacological strategies for the treatment of lymphedema caused by lymphatic injury during cancer treatment. They highlight the current treatment options for these patients, most of them being palliative in nature rather than considering the root cause of this disease. Traditional approaches rely mostly on compression garments and manual lymphatic drainage to decrease fluid accumulation. However, recent advances and discoveries in the field have identified potential alternatives, such as lymphangiogenic cytokine delivery, anti-inflammatory medications, and anti-fibrotic strategies. Likewise, these novel options may be used in combination with traditional methods or as adjuncts to surgical management.

Even if secondary lymphedema is usually a post-cancer treatment complication, common drugs administered to treat cardiovascular diseases and metabolic disorders can also promote the development of drug-induced lymphedema (Ridner and Dietrich, 2008). In their review article, Pal et al. summarize the evidences and potential mechanisms of drug-induced lymphedema. They describe how pharmacological regular medications for cardiovascular diseases, such as anti-arrhythmic agents (e.g., sodium, potassium and calcium channel blockers, and beta-adrenergic receptor blockers), vasodilators and K_ATP_ channel openers might contribute to the formation of lymphedema by impairing lymph flow as an off-target effect. Of note, calcium channel blockers are able to disrupt the cyclic contraction and relaxation of lymphatic vessels by modifying the physiological activity of calcium channels. The attenuation of the cyclic influx of calcium through L-type calcium channels in lymphatic smooth muscle cells impacts lymph flow and promotes the development of lymphedema. Following the same line, anti-diabetic agents such as the thiazidolinediones may also impair lymphatic function and contribute to the development of drug-induced secondary lymphedema. Additionally, chemotherapeutic agents such as anthracycline drugs (particularly doxorubicin), taxanes and corticosteroids co-administered with chemotherapy significantly contribute to lymphedema risk in cancer patients. However, on the other hand, other pharmacological treatments that include diuretics, hyaluronidase, ion channel modulating agents, anti-inflammatory and immunosuppressive agents are being administered to lymphedema patients with comorbid conditions to alleviate the symptoms of the disease. Up to date, there are no FDA-approved pharmacological agents to treat or prevent lymphedema. This work provides new guidance on potential therapies that may cause or reduce lymphedema, and therefore how patient medications can be managed to reduce lymphedema-related symptoms.

Clinical studies have shown that both, obesity and metabolic syndrome, are linked to lymphatic dysfunction and are a significant risk factor for the development of secondary lymphedema (Norden and Kume, 2020). Lee et al. review recent findings on collecting lymphatic vessel function and the reciprocal relationship between metabolic syndrome and lymphatic dysfunction. They also discuss how collecting lymphatic vessel contractile function can be improved by pharmacological approaches. Although important progress has been made in understanding the roles of the lymphatic system in metabolic diseases, still many questions remain to be answered, especially those related to the mechanisms driving changes in lymphatic contractile function. Up to date, only a few reports have investigated the molecular mechanisms and pathophysiological role of lymphatic contractile function in obesity or other metabolic disorders.

In the context of obesity and lymphatic vessels, Castorena-Gonzalez performed an interesting study that shows, for the first time, that lymphatic valve dysfunction could be a critical regulator of obesity-induced lymphedema, in association with the metabolic alterations induced by the western diet. To this end, Castorena used a diet-induced obesity model in mice and applied a methodology recently developed in his lab to quantitatively assess lymphatic valve function. First at all, obesity was induced in wild-type C57BL/6J mice by feeding a western diet for 14 weeks, which elevated plasma glucose and cholesterol levels when compared to controls. Then, valve function in isolated popliteal and mesenteric collecting lymphatic vessels from control and western diet-induced obese wild-type C57BL/6J mice was quantitatively assessed and compared, revealing that the function of lymphatic valves in popliteal lymphatics was not affected by diet-induced obesity, while back-leak of pressure, and leaky valves were detected in the mesenteric lymphatics. However, he also shows that globally deficient Plasminogen Activator Inhibitor-1 (PAI-1) mice, previously shown to be protected against the metabolic effects of a high fat diet, fed with a western diet were protected against metabolic dysfunction and displayed functional mesenteric lymphatic valves.

In addition to new potential roles of lymphatic vasculature dysfunction in obesity, the impact of lymphatic vessels in cancer immunotherapy is also gaining increasing attention (Lund et al., 2016). Interestingly, recent findings reveal that systemic anti-tumor immunity can be improved by targeting immune checkpoint therapy to tumor draining lymph nodes (Dammeijer et al., 2020). In this context, the role of T cells is well-described and tumor-associated dendritic cells display immune suppressive properties and induce tolerogenic T cells (Veglia and Gabrilovich, 2017). However, very little is known about B cell cancer immunity. The study by Louie et al. shows the crucial role of lymphatic function in the transport of tumor-associated antigens to B cells in the tumor draining lymph node to enhance B cell maturation. They also unravel how regional and systemic drug delivery to the tumor draining lymph nodes might affect the immune regulation. Louie and colleagues analyze how tumor growth reconstructs lymph node conduits and subcapsular sinus macrophages to ensure a more effective entry of tumor-associated antigens into the germinal center, thus promoting B cell activation and maturation. Furthermore, they find that regional delivery of clodronate liposome specifically depletes subcapsular sinus macrophages in the tumor draining lymph nodes, increases germinal center formation and induces tumor growth.

Overall, the articles building this special issue highlight the importance of the lymphatic vessels in health and disease, and how lymphatic vessel morphological or functional abnormalities are linked to numerous disorders. They also nicely summarize the current and potential future lymph-focused therapeutic approaches for lymphatic dysfunction and emphasize, not only the urgent need for developing more effective medical strategies to combat lymphatic dysfunction, but to careful evaluate the impact that regular pharmacological treatments for different pathologies could have on lymphatic function.

## References

[B1] Alitalo K. (2011). The Lymphatic Vasculature in Disease. Nat. Med. 17 (11), 1371–1380. 10.1038/nm.2545 22064427

[B2] Dammeijer F. van Gulijk M. Mulder E. E. Lukkes M. Klaase L. van den Bosch T. (2020). The PD-1/pd-L1-Checkpoint Restrains T Cell Immunity in Tumor-Draining Lymph Nodes. Cancer Cell 38 (5), 685–e8. 10.1016/j.ccell.2020.09.001 33007259

[B3] Lund A. W. Medler T. R. Leachman S. A. Coussens L. M. (2016). Lymphatic Vessels, Inflammation, and Immunity in Skin Cancer. Cancer Discov. 6 (1), 22–35. 10.1158/2159-8290.CD-15-0023 26552413 PMC4707138

[B4] Norden P. R. Kume T. (2020). The Role of Lymphatic Vascular Function in Metabolic Disorders. Front. Physiol. 11, 404. 10.3389/fphys.2020.00404 32477160 PMC7232548

[B5] Oliver G. Kipnis J. Randolph G. J. Harvey N. L. (2020). The Lymphatic Vasculature in the 21st Century: Novel Functional Roles in Homeostasis and Disease. Cell 182 (2), 270–296. 10.1016/j.cell.2020.06.039 32707093 PMC7392116

[B6] Ridner S. H. Dietrich M. S. (2008). Self-reported Comorbid Conditions and Medication Usage in Breast Cancer Survivors with and without Lymphedema. Oncol. Nurs. Forum 35 (1), 57–63. 10.1188/08.ONF.57-63 18192153

[B7] Rockson S. G. (2021). Advances in Lymphedema. Circ. Res. 128 (12), 2003–2016. 10.1161/CIRCRESAHA.121.318307 34110905

[B8] Veglia F. Gabrilovich D. I. (2017). Dendritic Cells in Cancer: the Role Revisited. Curr. Opin. Immunol. 45, 43–51. 10.1016/j.coi.2017.01.002 28192720 PMC5449252

